# Latent profiles of nutrition literacy among patients receiving peritoneal dialysis and their associations with dietary adherence and nutritional and metabolic indicators: a multicenter cross-sectional study

**DOI:** 10.3389/fpubh.2026.1935226

**Published:** 2026-10-14

**Authors:** Yanjing Chen, Shiyu Zhang, Caihua Ye, Minxiang Li, Xinjun Jiang

**Affiliations:** 1 School of Nursing, Hainan Medical University, Haikou, Hainan, China; 2 The Second Affiliated Hospital of Hainan Medical University, Haikou, Hainan, China

**Keywords:** dietary adherence, latent profile analysis, metabolic indicators, nutrition literacy, nutritional indicators, peritoneal dialysis

## Abstract

**Background:**

Nutrition literacy is important for dietary self-management among patients receiving peritoneal dialysis (PD), yet the heterogeneity of nutrition literacy and its associations with dietary adherence and nutritional and metabolic indicators remain poorly understood.

**Aims:**

To identify latent nutrition literacy profiles among patients receiving PD, examine factors associated with profile membership, and explore differences in dietary adherence and nutritional and metabolic indicators across profiles.

**Methods:**

A multicenter cross-sectional study was conducted among 307 patients receiving PD from three tertiary hospitals in Hainan Province, China, between May and December 2025. Data were collected using a demographic questionnaire, the Nutrition Literacy Scale for Patients with End-Stage Kidney Disease Undergoing Dialysis, and the Renal Adherence Attitudes Questionnaire and the Renal Adherence Behaviour Questionnaire. Nutritional and metabolic indicators were extracted from electronic medical records. Latent profile analysis was performed using Mplus 8.3, and group comparisons and multinomial logistic regression analyses were conducted using SPSS 27.0.

**Results:**

The mean nutrition literacy score was 14.10 ± 5.01. Three nutrition literacy profiles representing relatively low (46.5%), moderate (30.0%), and high (23.5%) levels were identified. Compared with the low profile, female sex and higher monthly personal income were associated with the moderate profile, while female sex, younger age, higher educational attainment, higher income, and longer dialysis duration were associated with the high profile (all *p* < 0.05). Differences across profiles remained statistically significant after false discovery rate correction for dietary adherence behavior and attitude scores and for several nutritional and metabolic indicators, including hemoglobin, serum albumin, lipid profiles, body mass index, and electrolyte levels (all adjusted *p* < 0.05).

**Conclusion:**

Nutrition literacy among patients receiving PD was heterogeneous, with three profiles differing in sociodemographic and disease-related characteristics, dietary adherence scores, and several nutritional and metabolic indicators. These profiles may provide a descriptive framework for recognizing subgroups with different nutrition support needs; however, their clinical utility requires external and longitudinal validation.

## 1 Introduction

Chronic kidney disease (CKD) has emerged as a major global public health challenge, with its prevalence and disease burden continuing to rise worldwide (1). As CKD progresses, a proportion of patients eventually develop end-stage kidney disease (ESKD) and require kidney replacement therapy to sustain life (2). Peritoneal dialysis (PD) is one of the major kidney replacement therapies for patients with ESKD and is widely used because of its home-based nature, cost-effectiveness, ease of administration, and ability to preserve residual kidney function (3).

Nevertheless, PD cannot fully replicate normal kidney function. During long-term treatment, patients frequently experience protein loss, toxin accumulation, electrolyte imbalances, and metabolic disturbances, which increase the risk of malnutrition and related complications (4). Previous studies have demonstrated that malnutrition is associated with impaired immune function, anemia, bone loss, reduced physical function, and poor clinical indicators (5, 6). Therefore, effective nutritional management is a cornerstone of PD care and plays a critical role in improving patients’ nutritional status and overall prognosis.

Unlike in-center hemodialysis, PD is primarily performed in the home setting and requires patients to independently manage complex dietary regimens over the long term. These regimens often involve balancing adequate protein intake with restrictions on sodium, phosphorus, potassium, and fluid consumption. Successful implementation of these recommendations depends not only on access to nutrition information but also on patients’ ability to understand, evaluate, and apply such information in daily life. Consequently, nutrition literacy may be associated with dietary self-management among patients receiving PD (7).

Nutrition literacy refers to an individual’s ability to obtain, understand, evaluate, and apply nutrition-related information to make appropriate dietary decisions (7). Higher levels of nutrition literacy may be associated with a greater capacity to interpret dietary recommendations, address nutrition-related problems, and apply nutrition knowledge in daily dietary practices (8). Dietary adherence may also be related to the association between nutrition literacy and health outcomes (9). Previous studies have shown that inadequate nutrition literacy may hinder patients’ ability to accurately understand complex dietary recommendations, including phosphorus restriction, sodium restriction, adequate intake of high-quality protein, and fluid control, thereby increasing the risk of malnutrition, hyperphosphataemia, dyslipidaemia, and electrolyte disturbances (10, 11).

Dietary self-management does not occur in isolation but forms part of the broader, multidomain self-management required of patients receiving PD. Physical activity represents another relevant lifestyle domain in this population and may interact with nutrition-related behaviors within patients’ broader patterns of functional health and daily disease management (12). Psychological well-being may influence patients’ capacity to undertake complex self-management tasks, whereas support from family members and healthcare professionals may facilitate access to health information and the maintenance of health-related behaviors (13). Among patients receiving PD, perceived social support has also been associated with both the physical and psychological dimensions of health-related quality of life (14). Collectively, these interrelated lifestyle and psychosocial domains may be associated with clinical status, quality of life, and overall well-being. However, how nutrition literacy and dietary self-management operate within this broader context remains insufficiently understood among patients receiving PD.

Against this broader background, existing studies of nutrition literacy have primarily focused on patients with diabetes, cardiovascular disease, or those undergoing hemodialysis, whereas evidence regarding patients receiving PD remains limited (15). Moreover, most previous studies have adopted variable-centered approaches based on total scores or mean values of nutrition literacy, implicitly assuming that patients constitute a relatively homogeneous population. However, patients receiving PD differ substantially in educational background, socioeconomic status, dialysis experience, self-management capacity, and nutritional risk. Patients with similar overall nutrition literacy scores may exhibit markedly different strengths and weaknesses across specific nutrition literacy domains. Such heterogeneity may be overlooked by traditional variable-centered approaches, limiting the identification of vulnerable subgroups and hindering the development of targeted nutrition management strategies (16).

More importantly, it remains unclear whether distinct nutrition literacy profiles are associated with differences in dietary adherence and objective nutritional and metabolic indicators among patients receiving PD. Characterizing these potential differences may help recognize subgroups with different nutrition support needs and inform subsequent clinical assessment.

To address these gaps, a person-centered analytical approach is needed to identify subgroups of patients who share similar nutrition literacy characteristics. Latent profile analysis (LPA) is a person-centered statistical approach that classifies individuals into distinct latent subgroups based on response patterns across multiple continuous indicators. Compared with traditional total-score analyses, LPA can uncover unobserved heterogeneity within populations and identify subgroups with distinct risk characteristics (17). Therefore, this study employed LPA to identify latent profiles of nutrition literacy among patients receiving PD, examine factors associated with profile membership, and explore the associations between different nutrition literacy profiles and dietary adherence, as well as nutritional and metabolic indicators. The findings may provide preliminary evidence for recognizing heterogeneity in nutrition literacy and informing future research on differentiated nutrition support for patients receiving PD.

## 2 Methods

### 2.1 Study design and participants

This multicenter cross-sectional study was reported in accordance with the Strengthening the Reporting of Observational Studies in Epidemiology (STROBE) guidelines (18). Patients receiving PD were recruited by convenience sampling from the nephrology departments of three tertiary hospitals in Haikou, Hainan Province, China, between May and December 2025.

Inclusion criteria were as follows: (1) diagnosis of end-stage kidney disease (19) and receipt of PD for at least 3 months; (2) age ≥18 years; (3) no cognitive, language, or communication impairments; and (4) willingness to participate and provision of written informed consent. Exclusion criteria were as follows: (1) severe psychiatric disorders, cognitive impairment, or language disorders; (2) concurrent hemodialysis treatment or previous kidney transplantation; (3) malignant tumors, active infections, tuberculosis, recent major surgery, or severe trauma; and (4) concurrent participation in other studies.

The minimum sample size was estimated using a commonly applied rule of thumb for regression analyses, which recommends approximately 10–15 participants per candidate predictor variable (20). For the 12 candidate variables examined in this study, the initial sample size requirement was 120–180 participants. After allowing for a 20% non-response or invalid-response rate, the minimum required sample size was adjusted to 150–225 participants. Given that adequate sample size is also important for the stability and interpretability of latent profile solutions, recruitment was not discontinued once this minimum was reached. Instead, recruitment continued throughout the prespecified study period, resulting in a final analytical sample of 307 participants.

### 2.2 Instruments

#### 2.2.1 Demographic questionnaire

A self-designed questionnaire was used to collect demographic and clinical information. Demographic variables included age, sex, occupation, educational background, marital status, monthly personal income, and medical expense coverage. Clinical data were extracted from the hospital electronic medical record system using information from the participants’ most recent outpatient or inpatient visit during the study period. Nutritional and metabolic indicators were extracted from electronic medical records, including hemoglobin (Hb), serum albumin (Alb), body mass index (BMI), total cholesterol (TC), triglycerides (TG), high-density lipoprotein cholesterol (HDL-C), low-density lipoprotein cholesterol (LDL-C), serum calcium (Ca), serum phosphorus (P), serum potassium (K), serum sodium (Na), serum creatinine (Scr), blood urea nitrogen (BUN), and estimated glomerular filtration rate (eGFR).

#### 2.2.2 Nutrition literacy scale for patients with end-stage kidney disease undergoing dialysis

The nutrition literacy scale for patients with end-stage kidney disease undergoing dialysis was developed by Li et al. (21) and published in 2023. The scale used in the present study comprised 25 dichotomously scored items across four dimensions: nutrition knowledge (9 items), cognitive attitude (5 items), behavioral practice (6 items), and information acquisition ability (5 items). Each item was scored as 0 or 1, yielding a total score ranging from 0 to 25, with higher scores indicating higher nutrition literacy. As no clinically validated cut-off has been established for defining adequate nutrition literacy within this scoring range, the total score was interpreted as reflecting the relative level of nutrition literacy rather than categorizing participants as having adequate or inadequate nutrition literacy. To capture heterogeneity across the four dimensions of nutrition literacy, the four dimension scores were entered as continuous indicators in the latent profile analysis to identify empirically derived profiles. Previous research reported an overall Cronbach’s α coefficient of 0.827, with dimension-specific coefficients ranging from 0.791 to 0.865. In the present study, the overall Cronbach’s α coefficient was 0.791.

#### 2.2.3 Renal adherence questionnaires

The Renal Adherence Questionnaires (RAQs), originally developed by Rushe and McGee (22) for patients receiving hemodialysis, consist of the Renal Adherence Attitudes Questionnaire (RAAQ) and the Renal Adherence Behaviour Questionnaire (RABQ). Both questionnaires have subsequently been used among Chinese patients receiving PD, including in the study by Gao et al. (23).

The RAAQ comprises four dimensions: attitudes toward social restrictions, health, self-care, and acceptance. It contains 26 items, each scored on a 5-point Likert scale ranging from 1 (“never”) to 5 (“always”). The total score ranges from 26 to 130, with higher scores indicating better adherence. Previous studies reported Cronbach’s α coefficients ranging from 0.68 to 0.88 across dimensions. In the present study, the overall Cronbach’s α coefficient was 0.905.

The RABQ comprises five dimensions: adherence to fluid restrictions, potassium and phosphate restrictions, self-care, sodium intake, and adherence in times of particular difficulty. It contains 25 items, each rated on a 5-point Likert scale ranging from 1 (“never”) to 5 (“always”), yielding a total score of 25–125, with higher scores indicating better adherence. In the present study, Cronbach’s α coefficients for the five dimensions ranged from 0.56 to 0.80, whereas the overall Cronbach’s α coefficient was 0.931. No dimensions or items were excluded, and the dimension scores were not analyzed separately; all analyses were based on the 25-item total score.

### 2.3 Data collection and quality control

To minimize selection and information bias, all investigators received standardized training before data collection and followed a uniform survey protocol. Five trained researchers screened hospital records to identify eligible participants according to the predefined inclusion and exclusion criteria. Eligible patients were informed about the purpose, content, and significance of the study, and written informed consent was obtained prior to participation.

Questionnaires were administered and collected on site and were completed independently by participants whenever possible. For participants who were unable to complete the questionnaire independently because of age, educational level, or physical limitations, trained researchers provided objective assistance in accordance with standardized procedures without offering guidance, suggestions, or leading responses. Completed questionnaires were reviewed immediately for completeness and plausibility, and any missing or unclear responses were verified with the participant at the time of collection.

To ensure data quality, two researchers independently entered the data into EpiData version 3.1, followed by consistency checks, logical validation, and error correction. Of the 320 questionnaires distributed, 315 were returned, yielding a response rate of 98.4%. Eight questionnaires with missing data on key study variables were excluded during data-quality screening, resulting in 307 complete questionnaires being included in the final analyses.

### 2.4 Statistical analysis

Statistical analyses were performed using SPSS version 27.0 and Mplus version 8.3, while the bootstrap validation was implemented in Python version 3.12. A two-sided significance level of α = 0.05 was adopted.

(1) Descriptive statistics: The Shapiro–Wilk test was used to assess the normality of continuous variables. Normally distributed continuous variables are presented as mean ± standard deviation (SD), whereas categorical variables are expressed as frequencies and percentages [*n* (%)].

(2) Latent profile analysis: LPA was conducted using Mplus version 8.3. Given the established four-dimensional structure of the Nutrition Literacy Scale for Patients with End-Stage Kidney Disease Undergoing Dialysis, scores for nutrition knowledge, cognitive attitude, behavioral practice, and information acquisition ability were entered as continuous indicators. Models containing one to five profiles were sequentially estimated using maximum likelihood estimation. The standard local-independence specification was applied, with within-profile indicator co-variances fixed at zero and indicator variances constrained to be equal across profiles. Multiple random starts were used, and replication of the best log-likelihood value was confirmed to reduce the risk of retaining a local maximum. Model fit was evaluated using the Akaike information criterion (AIC), Bayesian information criterion (BIC), sample-size-adjusted Bayesian information criterion (aBIC), entropy, the Lo–Mendell–Rubin likelihood ratio test (LMR), and the bootstrap likelihood ratio test (BLRT). Lower AIC, BIC, and aBIC values indicated better relative fit, whereas entropy values closer to 1 indicated greater classification accuracy. Significant LMR and BLRT results indicated that a model provided a better fit than the model with one fewer profile. The optimal solution was selected by jointly considering the fit indices, likelihood-ratio tests, classification accuracy, profile sizes, substantive interpretability, and model parsimony.

The stability of the selected solution was further evaluated using non-parametric bootstrap validation with 1,000 resamples drawn with replacement from the original sample. The three-profile model was refitted to each bootstrap sample using the same specification as in the primary analysis. Stability was assessed according to model convergence, replication of the ordered profile pattern across the four nutrition literacy dimensions, entropy, profile proportions, and agreement between the bootstrap-derived classifications and the original profile assignments.

(3) Intergroup comparisons: The chi-square test or Fisher’s exact test was used to compare categorical demographic and clinical characteristics across the nutrition literacy profiles. Normally distributed continuous outcomes were compared using one-way analysis of variance (ANOVA). Given the number of outcomes examined, the Benjamini–Hochberg procedure was applied separately to the dietary adherence behavior and attitude scores and the nutritional and metabolic indicators to control the false discovery rate. For outcomes remaining significant after this correction, *post hoc* pairwise comparisons were performed using the Bonferroni adjustment. Effect sizes for overall group differences were expressed as *η*^2^ with 95% confidence intervals (CIs). Statistical significance was determined using the false-discovery-rate-adjusted *p* value. As a sensitivity analysis, the Bolck–Croon–Hagenaars (BCH) auxiliary-variable approach was used to examine whether differences in continuous dietary adherence and nutritional and metabolic indicators across the latent profiles were robust to latent-profile classification uncertainty.

(4) Analysis of factors associated with profile membership: Multinomial logistic regression analysis was performed to examine factors associated with nutrition literacy profile membership, with the low nutrition literacy profile serving as the reference group. Variables that were statistically significant in univariate analyses and considered clinically relevant based on previous literature were entered into the model. The final model adjusted for sex, age, educational background, monthly personal income, number of comorbidities, and dialysis duration. Odds ratios (ORs) and 95% confidence intervals (CIs) were calculated.

(5) Missing-data analysis: Given that 2.5% of returned questionnaires contained missing data on key study variables, complete-case analysis was performed without multiple imputation. To assess potential selection bias associated with complete-case inclusion, the available demographic and clinical characteristics of participants included in and excluded from the final analyses were compared. Fisher’s exact test was used for categorical variables because of the small number of excluded participants.

## 3 Results

### 3.1 Demographic and clinical characteristics of patients receiving PD

Among the 307 patients receiving PD, 180 (58.6%) were male and 127 (41.4%) were female. The largest age group was 50–65 years, comprising 166 patients (54.1%). Farmers accounted for the highest proportion of occupations (57.0%, *n* = 175), and most participants were married (87.6%, *n* = 269). Educational background was generally low, with 84 patients (27.4%) having completed primary school or below and 124 (40.4%) having completed junior secondary school. Nearly half of the participants reported a monthly personal income of 2,000–5,000 yuan (49.2%), and the predominant form of medical expense coverage was the Basic Medical Insurance for Urban and Rural Residents (63.2%). Regarding disease-related characteristics, glomerular disease was the most common primary diagnosis (51.8%, *n* = 159). Most patients had three or more comorbidities (87.6%, *n* = 269), and 208 patients (67.8%) had undergone PD for ≤36 months (Table 1). Sensitivity analysis showed no statistically significant differences in the available demographic or clinical characteristics between participants included in and excluded from the final analyses (all *p* > 0.05) (Supplementary Table S1). These findings should be interpreted cautiously because only eight participants were excluded.

**Table 1:** Demographic and clinical characteristics of patients receiving PD (*N* = 307).

Variable	Category	Total, *n* (%)
Sex	Male	180 (58.6)
	Female	127 (41.4)
Age group (years)	18–34	39 (12.7)
	35–49	102 (33.2)
	50–65	166 (54.1)
Occupation	Farmer	175 (57.0)
	Employed	65 (21.2)
	Retired	61 (19.9)
	Student	6 (2.0)
Marital status	Single	35 (11.4)
	Married	269 (87.6)
	Divorced	3 (1.0)
Educational background	Primary school or below	84 (27.4)
	Junior secondary school	124 (40.4)
	Senior secondary school or vocational school	54 (17.6)
	College diploma	24 (7.8)
	Bachelor’s degree or above	21 (6.8)
Monthly personal income (yuan)	<2000	74 (24.1)
	2000–5,000	151 (49.2)
	>5,000	82 (26.7)
Medical expense coverage	Basic Medical Insurance for Urban and Rural Residents	194 (63.2)
	Basic Medical Insurance for Urban Employees	113 (36.8)
Primary diagnosis	Glomerular disease	159 (51.8)
	Diabetic kidney disease	45 (14.7)
	Hypertensive kidney damage	24 (7.8)
	Other	79 (25.7)
Number of comorbidities	≤1	8 (2.6)
	2	30 (9.8)
	≥3	269 (87.6)
Dialysis duration (months)	≤36	208 (67.8)
	37–71	81 (26.4)
	≥72	18 (5.9)

Data are presented as *n* (%). Percentages may not total 100.0% because of rounding. PD, peritoneal dialysis.

### 3.2 Current status of nutrition literacy among patients receiving PD

Among the 307 patients receiving PD, the mean total nutrition literacy score was 14.10 ± 5.01, with scores ranging from 3 to 25. Scores for the dimensions of nutrition knowledge, cognitive attitude, behavioral practice, and information acquisition ability were 5.19 ± 1.71, 2.72 ± 1.12, 3.44 ± 1.24, and 2.75 ± 1.16, respectively (Supplementary Table S2).

### 3.3 Latent profile analysis of nutrition literacy among patients receiving PD

Latent profile models containing one to five profiles were sequentially estimated using the four nutrition literacy dimension scores as manifest indicators. As the number of profiles increased, the AIC, BIC, and aBIC values progressively decreased. Both the LMR and BLRT were statistically significant for the two- and three-profile solutions, and the three-profile solution had an entropy value of 0.945, indicating high classification accuracy (Table 2).

**Table 2:** Fit indices for latent profile models of nutrition literacy among patients receiving PD.

Number of profiles	AIC	BIC	aBIC	Entropy	LMR *p*-value	BLR *P*-value	Profile proportions
1	4116.380	4146.195	4120.822	–	–	–	1.000
2	3194.508	3242.957	3201.727	0.916	<0.001	<0.001	0.577/0.423
3	2811.193	2878.277	2821.188	0.945	<0.001	<0.001	0.465/0.300/0.235
4	2579.728	2665.446	2592.500	0.993	0.055	<0.001	0.261/0.117/0.358/0.264
5	1903.777	2008.129	1919.325	0.992	0.717	<0.001	0.358/0.121/0.257/0.065/0.199

AIC, Akaike information criterion; BIC, Bayesian information criterion; aBIC, sample-size-adjusted Bayesian information criterion; LMR, Lo–Mendell–Rubin adjusted likelihood ratio test; BLRT, bootstrap likelihood ratio test; PD, peritoneal dialysis.

Although the AIC, BIC, and aBIC values decreased further for the four- and five-profile solutions, the LMR test did not indicate a statistically significant improvement over the preceding model for either the four-profile solution (*p* = 0.055) or the five-profile solution (*p* = 0.717). Moreover, the five-profile solution included a small profile comprising only 6.5% of the sample. Given that the additional profiles were not consistently supported by the likelihood-ratio tests and increased model complexity without sufficient statistical or substantive justification, the three-profile solution was retained on the basis of classification accuracy, profile sizes, substantive interpretability, and model parsimony. The three profiles comprised 46.5, 30.0, and 23.5% of the sample, respectively.

The stability of the selected model was further evaluated using 1,000 non-parametric bootstrap resamples. The three-profile model converged successfully in all resamples, and the ordered profile pattern was reproduced across all four nutrition literacy dimensions in every resample. The median bootstrap profile proportions were 46.0% (IQR: 43.8–48.2%), 29.7% (IQR: 27.8–31.9%), and 24.3% (IQR: 22.5–26.1%), respectively. The median entropy was 0.948 (IQR: 0.940–0.956), and the median classification agreement with the original solution was 99.0% (IQR: 98.4–99.7%). Overall, 96.0% of the bootstrap solutions achieved at least 90% classification agreement, supporting the stability of the selected three-profile solution.

### 3.4 Characteristics and naming of nutrition literacy profiles among patients receiving PD

The three nutrition literacy profiles were labeled according to their relative scores across the four dimensions of nutrition knowledge, cognitive attitude, behavioral practice, and information acquisition ability. As shown in Figure 1, the profiles followed a broadly parallel gradient across all four dimensions and therefore represented empirically derived low, moderate, and high levels of nutrition literacy rather than qualitatively contrasting dimensional patterns.

**Figure 1:**
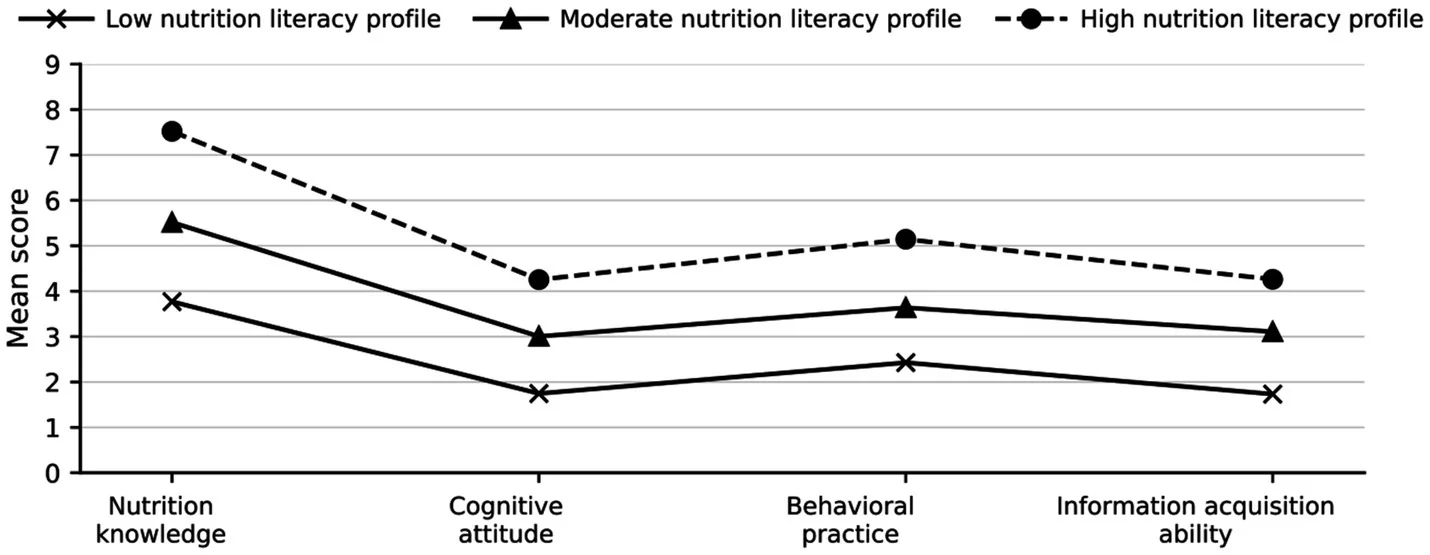
Dimension-specific score patterns across the three nutrition literacy profiles among patients receiving PD.

Profile 1, designated as the low nutrition literacy profile, included 143 participants (46.5%). Individuals in this profile exhibited relatively low scores across all four dimensions of nutrition literacy, namely nutrition knowledge, cognitive attitude, behavioral practice, and information acquisition ability.

Profile 2, designated as the moderate nutrition literacy profile, included 92 participants (30.0%). Participants in this profile demonstrated intermediate scores across all four dimensions.

Profile 3, designated as the high nutrition literacy profile, included 72 participants (23.5%). Participants in this profile exhibited consistently high scores across all four dimensions of nutrition literacy (Figure 1).

### 3.5 Univariate analysis of factors associated with nutrition literacy profiles among patients receiving PD

Chi-square tests or Fisher’s exact tests were used to compare demographic, socioeconomic, and clinical characteristics across the three nutrition literacy profiles. Significant differences were observed among the three profiles with respect to sex, age, educational background, monthly personal income, number of comorbidities, and dialysis duration (all *p* < 0.001). No significant differences were found in occupation, marital status, medical expense coverage, or primary diagnosis (all *p* > 0.05; Supplementary Table S3).

### 3.6 Multinomial logistic regression analysis of factors associated with nutrition literacy profiles among patients receiving PD

Using the low nutrition literacy profile as the reference group, variables that were statistically significant in the univariate analysis were entered into the multinomial logistic regression model. The coding of the independent variables is presented in Supplementary Table S4. Compared with the low nutrition literacy profile, female sex (OR = 2.014, 95% CI: 1.077–3.768) and higher monthly personal income (OR = 1.807, 95% CI: 1.139–2.866) were significantly associated with membership in the moderate nutrition literacy profile (both *p* < 0.05).

For the high nutrition literacy profile, female sex (OR = 2.817, 95% CI: 1.225–6.476), higher educational background (OR = 1.531, 95% CI: 1.013–2.313), higher monthly personal income (OR = 5.077, 95% CI: 2.572–10.022), and longer dialysis duration (OR = 3.402, 95% CI: 1.629–7.103) were positively associated with profile membership (all *p* < 0.05). In contrast, age was negatively associated with membership in the high nutrition literacy profile (OR = 0.492, 95% CI: 0.267–0.905, *p* = 0.022).

The number of comorbidities was not significantly associated with profile membership (*p* > 0.05; Table 3). Overall, female sex and higher monthly personal income were consistently associated with membership in the moderate and high nutrition literacy profiles.

**Table 3:** Multinomial logistic regression analysis of factors associated with nutrition literacy profile membership among patients receiving PD.

Variable	*B*	*SE*	Wald *χ*^2^	*P* value	aOR	95% CI
Moderate nutrition literacy profile vs. low nutrition literacy profile						
Intercept	−11.771	2.443	23.215	<0.001	–	–
Sex	0.700	0.320	4.800	0.028	2.014	1.077–3.768
Age group	−0.105	0.247	0.180	0.671	0.901	0.555–1.461
Educational background	0.214	0.170	1.586	0.208	1.239	0.888–1.728
Monthly personal income	0.592	0.235	6.324	0.012	1.807	1.139–2.866
Number of comorbidities	0.193	0.482	0.161	0.688	1.213	0.472–3.122
Dialysis duration	0.118	0.310	0.146	0.702	1.126	0.613–2.067
High nutrition literacy profile vs. low nutrition literacy profile						
Intercept	−25.408	3.852	43.514	<0.001	–	–
Sex	1.036	0.425	5.947	0.015	2.817	1.225–6.476
Age group	−0.710	0.311	5.209	0.022	0.492	0.267–0.905
Educational background	0.426	0.211	4.089	0.043	1.531	1.013–2.313
Monthly personal income	1.625	0.347	21.933	<0.001	5.077	2.572–10.022
Number of comorbidities	0.730	0.588	1.541	0.214	2.076	0.655–6.577
Dialysis duration	1.224	0.376	10.623	<0.001	3.402	1.629–7.103

The low nutrition literacy profile was used as the reference category. The model was adjusted for sex, age group, educational background, monthly personal income, number of comorbidities, and dialysis duration. aOR, adjusted odds ratio; CI, confidence interval; SE, standard error; PD, peritoneal dialysis.

### 3.7 Comparison of dietary adherence behavior and attitude scores across nutrition literacy profiles among patients receiving PD

After correction for multiple comparisons using the Benjamini–Hochberg procedure, differences across the nutrition literacy profiles remained significant for both dietary adherence behavior and attitude scores (both FDR-adjusted *p* < 0.001). The between-profile difference in dietary adherence behavior represented a moderate effect (*η*^2^ = 0.084, 95% CI: 0.032–0.146), whereas the difference in dietary adherence attitude represented a larger effect (*η*^2^ = 0.173, 95% CI: 0.101–0.247). Bonferroni-adjusted pairwise comparisons showed that the low nutrition literacy profile had lower behavior and attitude scores than both the moderate and high profiles (all adjusted *p* < 0.001). The moderate and high profiles differed in attitude scores (adjusted *p* < 0.001), but not in behavior scores (adjusted *p* = 0.993; Supplementary Table S5).

### 3.8 Comparison of nutritional and metabolic indicators across nutrition literacy profiles among patients receiving PD

After correction for multiple comparisons using the Benjamini–Hochberg procedure, significant differences across the nutrition literacy profiles remained for Hb, Alb, BMI, TC, TG, HDL-C, LDL-C, Ca, P, K, and Na. The FDR-adjusted *p*-value was 0.049 for K and <0.001 for the other indicators. Effect sizes for these significant differences ranged from *η*^2^ = 0.021 for K (95% CI: 0.000–0.059) to *η*^2^ = 0.341 for HDL-C (95% CI: 0.259–0.416). Relatively large between-profile differences were also observed for Na (*η*^2^ = 0.316, 95% CI: 0.233–0.391), TG (*η*^2^ = 0.289, 95% CI: 0.207–0.365), and Hb (*η*^2^ = 0.266, 95% CI: 0.185–0.342). No significant differences were observed in Scr, BUN, or eGFR (*η*^2^ = 0.002–0.014; Table 4).

**Table 4:** Comparison of nutritional and metabolic indicators across nutrition literacy profiles among patients receiving PD.

Variable	Low profile (*n* = 143)	Moderate profile (*n* = 92)	High profile (*n* = 72)	*F*	FDR-adjusted P	*η*^2^ (95% CI)	Bonferroni-adjusted pairwise *P*
Nutritional indicators							
Hb (g/L)	96.93 ± 13.25	108.04 ± 13.91	116.81 ± 13.61	55.143	<0.001	0.266 (0.185–0.342)	L–M < 0.001; L–H < 0.001; M–H < 0.001
Alb (g/L)	29.91 ± 3.72	32.42 ± 3.64	33.93 ± 3.43	32.720	<0.001	0.177 (0.104–0.251)	L–M < 0.001; L–H < 0.001; M–H 0.009
BMI (kg/m^2^)	24.56 ± 2.36	23.79 ± 2.11	23.03 ± 2.15	11.487	<0.001	0.070 (0.023–0.129)	L–M 0.011; L–H < 0.001; M–H 0.033
Lipid indicators							
TC (mmol/L)	4.37 ± 0.71	3.91 ± 0.66	3.51 ± 0.63	40.989	<0.001	0.212 (0.135–0.288)	L–M < 0.001; L–H < 0.001; M–H < 0.001
TG (mmol/L)	2.14 ± 0.70	1.66 ± 0.56	1.18 ± 0.45	61.749	<0.001	0.289 (0.207–0.365)	L–M < 0.001; L–H < 0.001; M–H < 0.001
HDL-C (mmol/L)	0.95 ± 0.16	1.06 ± 0.18	1.25 ± 0.15	78.746	<0.001	0.341 (0.259–0.416)	L–M < 0.001; L–H < 0.001; M–H < 0.001
LDL-C (mmol/L)	2.65 ± 0.59	2.45 ± 0.50	2.31 ± 0.56	9.990	<0.001	0.062 (0.017–0.118)	L–M 0.007; L–H < 0.001; M–H 0.109
Electrolyte indicators							
Ca (mmol/L)	2.14 ± 0.12	2.18 ± 0.11	2.21 ± 0.13	9.584	<0.001	0.059 (0.016–0.115)	L–M 0.014; L–H < 0.001; M–H 0.072
P (mmol/L)	1.69 ± 0.24	1.54 ± 0.20	1.42 ± 0.22	37.138	<0.001	0.196 (0.121–0.271)	L–M < 0.001; L–H < 0.001; M–H 0.001
K (mmol/L)	4.03 ± 0.38	3.99 ± 0.34	3.90 ± 0.33	3.285	0.049	0.021 (0.000–0.059)	L–M 0.423; L–H 0.011; M–H 0.096
Na (mmol/L)	134.03 ± 2.66	135.33 ± 2.82	138.57 ± 2.42	70.120	<0.001	0.316 (0.233–0.391)	L–M < 0.001; L–H < 0.001; M–H < 0.001
Renal function indicators							
Scr (μmol/L)	963.57 ± 306.29	988.12 ± 291.14	986.17 ± 268.67	0.250	0.779	0.002 (0.000–0.016)	–
BUN (mmol/L)	23.78 ± 8.04	25.30 ± 6.87	25.77 ± 6.87	2.136	0.140	0.014 (0.000–0.047)	–
eGFR (mL/min/1.73 m^2^)	11.62 ± 1.35	11.88 ± 1.38	11.50 ± 1.31	1.800	0.180	0.012 (0.000–0.042)	–

Data are presented as mean ± standard deviation. Overall differences were assessed using one-way analysis of variance. False-discovery-rate-adjusted p values were calculated across the 14 nutritional and metabolic indicators using the Benjamini–Hochberg procedure. Pairwise comparisons were conducted only for outcomes with a statistically significant overall FDR-adjusted p value, and the corresponding pairwise p values were adjusted using the Bonferroni method. Effect sizes are presented as η^2^ with 95% confidence intervals. Hb, hemoglobin; Alb, serum albumin; BMI, body mass index; TC, total cholesterol; TG, triglycerides; HDL-C, high-density lipoprotein cholesterol; LDL-C, low-density lipoprotein cholesterol; Ca, serum calcium; P, serum phosphorus; K, serum potassium; Na, serum sodium; Scr, serum creatinine; BUN, blood urea nitrogen; eGFR, estimated glomerular filtration rate; CI, confidence interval; FDR, false discovery rate; PD, peritoneal dialysis.

For the nutritional indicators, Hb and Alb levels increased across the low, moderate, and high nutrition literacy profiles, whereas BMI decreased. Bonferroni-adjusted pairwise comparisons showed that participants in the high nutrition literacy profile had higher Hb and Alb levels and lower BMI than those in the low nutrition literacy profile.

Differences were also observed in the lipid and electrolyte indicators. Compared with the low nutrition literacy profile, the high profile had lower TC, TG, and LDL-C levels, higher HDL-C levels, higher Ca and Na levels, and lower P and K levels. The effect sizes ranged from small for K (*η*^2^ = 0.021) to large for HDL-C (*η*^2^ = 0.341) and Na (*η*^2^ = 0.316). No significant differences were observed in Scr, BUN, or eGFR after correction for multiple comparisons.

The BCH sensitivity analyses yielded the same overall significance pattern as the analyses based on most-likely profile assignment, supporting the robustness of the observed between-profile differences.

## 4 Discussion

This study identified three empirically derived nutrition literacy profiles among patients receiving PD. The profiles exhibited a broadly parallel gradient across nutrition knowledge, cognitive attitude, behavioral practice, and information acquisition ability, indicating that they primarily represented relatively low, moderate, and high levels of nutrition literacy. Nearly half of the participants belonged to a consistently low-scoring profile across all four dimensions. The profiles also differed in sociodemographic and disease-related characteristics, dietary adherence scores, and several nutritional and metabolic indicators. Although the resulting profiles were primarily quantitative rather than qualitatively distinct, LPA provided information beyond the sample mean or an arbitrary total-score threshold by jointly considering the four dimension scores and empirically identifying subgroups without requiring a predefined clinical cut-off. This person-centered analysis therefore provided a descriptive framework for examining heterogeneity in nutrition literacy and the characteristics associated with different nutrition literacy levels.

This study found that the low nutrition literacy profile accounted for the largest proportion of the study population. Participants in this profile exhibited low scores across all four dimensions of nutrition literacy—nutrition knowledge, cognitive attitude, behavioral practice, and information acquisition ability. They were more likely to be male and older and to have lower educational attainment, lower personal income, and a shorter dialysis duration, consistent with previous studies (24, 25). These characteristics may reflect disparities in access to health information and self-management resources, which could be associated with lower nutrition literacy. In addition, these participants had lower dietary adherence behavior and attitude scores, suggesting that lower nutrition literacy may be accompanied by difficulties in translating nutrition-related knowledge into dietary self-management practices (17).

Furthermore, participants in this profile had the lowest Hb and Alb levels but the highest BMI among the three profiles. However, a higher BMI should not necessarily be interpreted as indicating better nutritional status in patients receiving PD. BMI may be confounded by fluid status because volume overload can increase body weight without reflecting a corresponding increase in adipose tissue or lean body mass (26). Peritoneal ultrafiltration volume and residual urine output are important determinants of volume status, whereas Kt/V provides an estimate of small-solute clearance and the amount of dialysis delivered (27). These dialysis-related parameters were not collected in the present study, limiting our ability to account for potential differences in dialysis adequacy and fluid status when interpreting the observed nutritional indicators. Therefore, the observed differences in BMI across the nutrition literacy profiles should be interpreted cautiously and in conjunction with other nutritional indicators. Future studies should incorporate dialysis adequacy and fluid removal parameters together with objective body composition assessments, such as bioelectrical impedance analysis performed under standardized conditions, to better distinguish fluid overload from adipose tissue and lean body mass.

The magnitude of several between-profile differences may also be clinically relevant. The mean serum Alb level in the low nutrition literacy profile was 29.91 g/L, compared with 33.93 g/L in the high profile. The mean value in the low profile was therefore below 30 g/L, suggesting that the observed difference may be clinically relevant and may warrant greater attention to nutritional risk in this group.

Nevertheless, serum Alb is also influenced by inflammation and volume status and should therefore be interpreted alongside dietary intake, body composition, and other nutritional assessments rather than as an independent diagnostic criterion for malnutrition (28). The mean serum P level was higher in the low profile than in the high profile (1.69 vs. 1.42 mmol/L), although neither group mean exceeded the upper recommended level of 1.78 mmol/L for patients receiving dialysis (29). Although statistically significant differences were also observed in Ca, K, and Na, these group-level differences do not necessarily indicate clinically meaningful electrolyte abnormalities or establish their prevalence within each profile. Electrolyte concentrations in patients receiving PD may also be associated with residual kidney function, dialysis prescription and adequacy, volume status, and medication use (30). These findings should therefore be interpreted cautiously and considered together with individual clinical assessments.

No significant differences in Scr, BUN, or eGFR were observed across the three nutrition literacy profiles. These kidney function indicators are associated with multiple clinical factors, including disease progression, residual kidney function, and dialysis-related characteristics. Therefore, the absence of between-profile differences in these indicators should not be interpreted as evidence that nutrition literacy is unrelated to kidney function. From a clinical perspective, the lower Hb and Alb levels, higher TC, TG, and LDL-C levels, and lower HDL-C level observed in the low nutrition literacy profile may be particularly relevant for clinical assessment, while the higher P level also warrants attention in the context of individual biochemical monitoring.

Together, these findings support comprehensive nutritional assessment, anemia evaluation, medication review, and ongoing biochemical monitoring for patients in this profile. Given that this profile accounted for the largest proportion of the sample and was characterized by consistently low nutrition literacy scores, patients in this group may represent a priority population for nutrition support. Interventions could prioritize strengthening basic nutrition knowledge and improving access to nutrition-related information. Previous studies have suggested that visual educational materials, practical demonstrations, and personalized guidance may support disease awareness and self-management abilities among patients (31, 32). Involving family members in disease management and providing digital health education may also support treatment adherence and patients’ ability to obtain and apply nutrition-related information (33, 34).

Participants in the moderate nutrition literacy profile demonstrated intermediate scores across nutrition knowledge, cognitive attitude, behavioral practice, and information acquisition ability. Their demographic and clinical characteristics generally occupied an intermediate position between those of the low and high profiles. Dietary adherence behavior and attitude scores, as well as Hb, Alb, and several metabolic indicators, were also generally intermediate. These findings suggest that individuals in this profile may possess basic nutrition-related knowledge but may experience difficulty translating that knowledge into consistent dietary self-management practices.

Previous studies have shown that behavioral strategies such as goal-setting, motivational interviewing, and shared decision-making may enhance patients’ self-efficacy, self-management skills, and long-term adherence (35, 36). In addition, social support approaches, including peer support, group-based interventions, and continuous feedback, have been associated with improved maintenance of healthy behaviors among individuals with chronic conditions such as diabetes and hypertension (37, 38). Accordingly, interventions targeting individuals in the moderate nutrition literacy profile should move beyond knowledge dissemination and place greater emphasis on behavior change. Particular attention should be directed toward helping patients translate existing nutrition-related knowledge into sustained health-promoting dietary behaviors and strengthening their long-term dietary self-management capabilities.

Participants in the high nutrition literacy profile demonstrated consistently high scores across all dimensions of nutrition literacy, including nutrition knowledge, cognitive attitude, behavioral practice, and information acquisition ability. Dietary adherence behavior and attitude scores were also highest in this profile, consistent with the observed differences in dietary adherence across the nutrition literacy profiles and with previous findings reported by McAuley et al. (39). Individuals in this profile were more likely to be female, younger, have higher educational attainment and personal income, and have a longer dialysis duration. These characteristics may reflect differences in access to and utilization of nutrition-related information, dietary behaviors, and nutritional self-management. The observed association between longer dialysis duration and higher nutrition literacy may reflect greater cumulative exposure to nutrition education and more frequent interactions with healthcare professionals during long-term PD treatment, although these potential explanations were not directly examined in the present study.

Regarding nutritional and metabolic indicators, participants in the high nutrition literacy profile had relatively higher Hb and Alb levels and lower BMI values than those in the low profile. They also had lower TC, TG, and LDL-C levels and higher HDL-C levels, while differences were observed in several electrolyte indicators, including Ca, P, K, and Na. Collectively, these findings indicate that the high nutrition literacy profile was characterized by a distinct pattern of nutritional and metabolic indicators. However, given the cross-sectional design and the potential role of dialysis-related and other clinical factors, these profile-specific differences should not be interpreted as indicating an overall more favorable clinical status.

Nutrition-related behaviors should also be interpreted within the broader, multidomain context of self-management among patients receiving PD. Dietary practices may operate alongside physical activity, as both domains contribute to patients’ functional health and capacity for daily disease management (40). Psychological distress and reduced emotional well-being may also be relevant to patients’ motivation and capacity to sustain complex dietary and other treatment-related routines (41). At the same time, support from family members and healthcare professionals may help patients access, interpret, and apply nutrition information within their everyday lives. These interrelated lifestyle and psychosocial domains may be relevant to patients’ self-management experiences, clinical status, health-related quality of life, and overall well-being. However, physical activity, psychological well-being, social support, and quality of life were not directly assessed in the present study; therefore, their relationships with the identified nutrition literacy profiles could not be examined. Future research should incorporate these dimensions and evaluate integrated, person-centered interventions that combine nutrition literacy support with physical activity promotion, psychological care, and family or professional support (42). Such interventions should be adapted to patients’ individual needs and evaluated using both clinical indicators and patient-reported outcomes, including health-related quality of life and psychological well-being.

Although the three profiles exhibited an ordered gradient from low to moderate and high nutrition literacy, they should not be interpreted as established developmental stages. The cross-sectional LPA identified subgroups at a single time point and could not determine whether individual patients progress sequentially between profiles. Nevertheless, profile membership may provide a useful framework for considering the timing and intensity of nutrition support according to patients’ current needs. From a clinical perspective, patients currently classified in the low profile may warrant earlier and more intensive support focused on foundational nutrition knowledge and access to nutrition information; those in the moderate profile may benefit from timely behavioral reinforcement aimed at translating existing knowledge into sustained dietary practices; and those in the high profile may be considered for maintenance-oriented support, regular nutritional monitoring, and personalized follow-up (43). Longitudinal studies with repeated assessments, potentially using latent transition analysis, are needed to evaluate profile stability and transitions over time and to determine whether tailored interventions are associated with movement between profiles.

### 4.1 Limitations and recommendations

This study employed LPA to identify nutrition literacy profiles among patients receiving PD and to examine their associations with dietary adherence and nutritional and metabolic indicators. The findings may inform the development of stratified nutrition support for this population. Nevertheless, several limitations should be acknowledged.

First, the cross-sectional design precludes causal inference and does not allow the temporal relationships among nutrition literacy, dietary adherence, and nutritional and metabolic indicators to be established. Longitudinal studies are therefore needed to examine changes in nutrition literacy profiles over time and clarify the temporal relationships among these variables.

Second, although the multicenter design increased sample diversity, all participants were recruited by convenience sampling from three tertiary hospitals in Hainan Province. The relatively high proportions of farmers and participants with low educational attainment may reflect the local sociodemographic structure and may be associated with differences in access to nutrition information, dietary practices, and self-management resources. Recruitment from tertiary hospitals may also have underrepresented patients receiving care in secondary hospitals or community-based PD programs. Although heterogeneity in nutrition literacy may also exist in other populations, the number, size, and characteristics of the identified profiles may differ across geographic regions, countries, and healthcare systems. Differences in the availability of renal dietetic services, the delivery of PD education and follow-up, and access to health information may further affect the applicability of these findings. External validation in populations with more diverse socioeconomic, geographic, cultural, and healthcare backgrounds is therefore warranted.

Third, the dichotomous item scoring of the Nutrition Literacy Scale may have reduced its sensitivity to subtle differences in participants’ responses and limited the precision with which more nuanced nutrition literacy profiles could be distinguished. Future studies should examine whether instruments with polytomous response options provide greater sensitivity for identifying nutrition literacy patterns. In addition, although the RAAQ and RABQ demonstrated high overall internal consistency in the present study, Cronbach’s α coefficients below 0.70 for some RABQ dimensions indicated limited reliability at the subscale level. The reliability of individual RABQ subscales should therefore be interpreted cautiously, although the present analyses were based on the total score rather than individual dimension scores. Dedicated psychometric validation of both questionnaires among Chinese patients receiving PD remains limited, and further evaluation of their reliability and validity in this population is warranted.

Fourth, the between-profile comparisons of nutritional and metabolic indicators were not adjusted for potentially relevant clinical factors. Although the multinomial logistic regression examining profile membership adjusted for sex, age, educational background, monthly personal income, number of comorbidities, and dialysis duration, this adjustment did not extend to the biochemical comparisons. Several potentially relevant clinical variables were also unavailable, including dialysis adequacy, residual kidney function, ultrafiltration and volume status, history of peritonitis, inflammatory status, and the use of medications or dietary supplements that may affect nutritional and metabolic indicators. In addition, physical activity, psychological well-being, social support, and health-related quality of life were not directly assessed; therefore, their relationships with nutrition literacy profiles and dietary self-management could not be examined. This limited our ability to situate the identified profiles within the broader multidomain context of lifestyle, self-management, and well-being. Future studies should prospectively assess these lifestyle and psychosocial factors and incorporate relevant covariates and patient-reported outcomes into multivariable analyses examining profile membership, dietary self-management, and clinical indicators.

Finally, the study was not specifically designed or powered to evaluate effect modification by educational attainment or dialysis duration, and some combinations of these variables with nutrition literacy profiles contained very small numbers of participants. Interaction analyses were therefore not performed because the resulting estimates could be unstable. Future studies with larger and more diverse samples should examine whether educational attainment or dialysis duration modifies the associations between nutrition literacy profiles and dietary adherence or nutritional and metabolic indicators.

## 5 Conclusion

Three empirically derived nutrition literacy profiles were identified among patients receiving PD, with distinct patterns of sociodemographic and disease-related characteristics, dietary adherence scores, and several nutritional and metabolic indicators. These findings highlight the heterogeneity of nutrition literacy and its associations with dietary and clinical characteristics in this population. The identified profiles may provide a descriptive framework for recognizing patients with different nutrition support needs, although their clinical utility requires confirmation in external and longitudinal studies.

## Data Availability

The original contributions presented in the study are included in the article/Supplementary material, further inquiries can be directed to the corresponding author.
