# Supplementary material for: Latent profiles of nutrition literacy among patients receiving peritoneal dialysis and their associations with dietary adherence and nutritional and metabolic indicators: a multicenter cross-sectional study
Source: Front Public Health. 2026 Oct 14;14:1935226. doi: 10.3389/fpubh.2026.1935226 (PMC13713849; doi:10.3389/fpubh.2026.1935226)
Supplement: Supplementary file 1 [file Table_1.DOCX]

**Supplementary Table S1. Comparison of Available Demographic and Clinical Characteristics Between Participants Included in and Excluded From the Final Analyses**

| **Characteristic** | **Included (n=307)** | **Excluded (n=8)** | ***P* value** |
| --- | --- | --- | --- |
| **Sex** |  |  | 1.000 |
| Male | 180 (58.6) | 5 (62.5) |  |
| Female | 127 (41.4) | 3 (37.5) |  |
| **Age group (years)** |  |  | 1.000 |
| 18–34 | 39 (12.7) | 1 (12.5) |  |
| 35–49 | 102 (33.2) | 3 (37.5) |  |
| 50–65 | 166 (54.1) | 4 (50.0) |  |
| **Occupation** |  |  | 0.830 |
| Farmer | 175 (57.0) | 4 (50.0) |  |
| Employed | 65 (21.2) | 2 (25.0) |  |
| Retired | 61 (19.9) | 2 (25.0) |  |
| Student | 6 (2.0) | 0 (0.0) |  |
| **Marital status** |  |  | 1.000 |
| Single | 35 (11.4) | 1 (12.5) |  |
| Married | 269 (87.6) | 7 (87.5) |  |
| Divorced | 3 (1.0) | 0 (0.0) |  |
| **Educational background** |  |  | 0.766 |
| Primary school or below | 84 (27.4) | 2 (25.0) |  |
| Junior secondary school | 124 (40.4) | 3 (37.5) |  |
| Senior secondary or vocational school | 54 (17.6) | 1 (12.5) |  |
| College diploma | 24 (7.8) | 1 (12.5) |  |
| Bachelor’s degree or above | 21 (6.8) | 1 (12.5) |  |
| **Monthly personal income (yuan)** |  |  | 1.000 |
| <2,000 | 74 (24.1) | 2 (25.0) |  |
| 2,000–5,000 | 151 (49.2) | 4 (50.0) |  |
| >5,000 | 82 (26.7) | 2 (25.0) |  |
| **Medical expense coverage** |  |  | 1.000 |
| Basic Medical Insurance for Urban and Rural Residents | 194 (63.2) | 5 (62.5) |  |
| Basic Medical Insurance for Urban Employees | 113 (36.8) | 3 (37.5) |  |
| **Primary diagnosis** |  |  | 0.885 |
| Glomerular disease | 159 (51.8) | 4 (50.0) |  |
| Diabetic kidney disease | 45 (14.7) | 1 (12.5) |  |
| Hypertensive kidney damage | 24 (7.8) | 1 (12.5) |  |
| Other | 79 (25.7) | 2 (25.0) |  |
| **Number of comorbidities** |  |  | 0.655 |
| ≤1 | 8 (2.6) | 0 (0.0) |  |
| 2 | 30 (9.8) | 1 (12.5) |  |
| ≥3 | 269 (87.6) | 7 (87.5) |  |
| **Dialysis duration (months)** |  |  | 0.530 |
| ≤36 | 208 (67.8) | 5 (62.5) |  |
| 37–71 | 81 (26.4) | 2 (25.0) |  |
| ≥72 | 18 (5.9) | 1 (12.5) |  |

**Note:** Data are presented as n (%). Fisher’s exact test was used for categorical variables because of the small number of excluded participants. Only variables with available data in both groups were compared.

**Supplementary Table S2. Nutrition Literacy Scores Among Patients Receiving PD (N = 307)**

| **Variable** | **Theoretical score range** | **Observed range** | **Mean ± SD** |
| --- | --- | --- | --- |
| Total nutrition literacy score | 0–25 | 3–25 | 14.10 ± 5.01 |
| Nutrition knowledge | 0–9 | 2–9 | 5.19 ± 1.71 |
| Cognitive attitude | 0–5 | 0–5 | 2.72 ± 1.12 |
| Behavioral practice | 0–6 | 1–6 | 3.44 ± 1.24 |
| Information acquisition ability | 0–5 | 0–5 | 2.75 ± 1.16 |

**Note:** Data are presented as mean ± standard deviation (SD). The observed range represents the minimum and maximum scores recorded in the study sample. PD, peritoneal dialysis.

**Supplementary Table S3. Univariate Analysis of Factors Associated With Nutrition Literacy Profiles Among Patients Receiving PD (N = 307)**

| **Variable** | **Category** | **Low nutrition literacy profile** **(n = 143)** | **Moderate nutrition literacy profile** **(n = 92)** | **High nutrition literacy profile** **(n = 72)** | **Test statistic** | ***P* value** |
| --- | --- | --- | --- | --- | --- | --- |
| **Sex** | Male | 110 (76.9) | 47 (51.1) | 23 (31.9) | 43.026^a^ | <0.001 |
|  | Female | 33 (23.1) | 45 (48.9) | 49 (68.1) |  |  |
| **Age group (years)** | 18–34 | 6 (4.2) | 11 (12.0) | 22 (30.6) | 34.355^a^ | <0.001 |
|  | 35–49 | 48 (33.6) | 28 (30.4) | 26 (36.1) |  |  |
|  | 50–65 | 89 (62.2) | 53 (57.6) | 24 (33.3) |  |  |
| **Occupation** | Farmer | 80 (55.9) | 56 (60.9) | 39 (54.2) | 4.811^b^ | 0.568 |
|  | Employed | 29 (20.3) | 15 (16.3) | 21 (29.2) |  |  |
|  | Retired | 31 (21.7) | 19 (20.7) | 11 (15.3) |  |  |
|  | Student | 3 (2.1) | 2 (2.2) | 1 (1.4) |  |  |
| **Marital status** | Single | 16 (11.2) | 9 (9.8) | 10 (13.9) | 2.032^b^ | 0.730 |
|  | Married | 125 (87.4) | 83 (90.2) | 61 (84.7) |  |  |
|  | Divorced | 2 (1.4) | 0 (0.0) | 1 (1.4) |  |  |
| **Educational background** | Primary school or below | 48 (33.6) | 23 (25.0) | 13 (18.1) | 58.560^a^ | <0.001 |
|  | Junior secondary school | 70 (49.0) | 33 (35.9) | 21 (29.2) |  |  |
|  | Senior secondary school or vocational school | 19 (13.3) | 25 (27.2) | 10 (13.9) |  |  |
|  | College diploma | 4 (2.8) | 7 (7.6) | 13 (18.1) |  |  |
|  | Bachelor's degree or above | 2 (1.4) | 4 (4.3) | 15 (20.8) |  |  |
| **Monthly personal income (yuan)** | <2000 | 49 (34.3) | 21 (22.8) | 4 (5.6) | 44.841^a^ | <0.001 |
|  | 2000–5000 | 76 (53.1) | 44 (47.8) | 31 (43.1) |  |  |
|  | >5000 | 18 (12.6) | 27 (29.3) | 37 (51.4) |  |  |
| **Medical expense coverage** | Basic Medical Insurance for Urban and Rural Residents | 91 (63.6) | 63 (68.5) | 40 (55.6) | 2.923^a^ | 0.232 |
|  | Basic Medical Insurance for Urban Employees | 52 (36.4) | 29 (31.5) | 32 (44.4) |  |  |
| **Primary diagnosis** | Glomerular disease | 69 (48.3) | 50 (54.3) | 40 (55.6) | 4.345^a^ | 0.630 |
|  | Diabetic kidney disease | 23 (16.1) | 15 (16.3) | 7 (9.7) |  |  |
|  | Hypertensive kidney damage | 13 (9.1) | 4 (4.3) | 7 (9.7) |  |  |
|  | Other | 38 (26.6) | 23 (25.0) | 18 (25.0) |  |  |
| **Number of comorbidities** | ≤1 | 0 (0.0) | 1 (1.1) | 7 (9.7) | 24.959^b^ | <0.001 |
|  | 2 | 20 (14.0) | 8 (8.7) | 2 (2.8) |  |  |
|  | ≥3 | 123 (86.0) | 83 (90.2) | 63 (87.5) |  |  |
| **Dialysis duration (months)** | ≤36 | 111 (77.6) | 64 (69.6) | 33 (45.8) | 41.117^b^ | <0.001 |
|  | 37–71 | 29 (20.3) | 27 (29.3) | 25 (34.7) |  |  |
|  | ≥72 | 3 (2.1) | 1 (1.1) | 14 (19.4) |  |  |

**Note:** Data are presented as n (%), with percentages calculated within each nutrition literacy profile and reported to one decimal place. Cells reported as 0 (0.0) represent observed zero counts rather than missing data. ᵃ Pearson’s chi-square test. ᵇ Fisher’s exact test.

**Supplementary Table S4. Coding of Independent Variables Included in the Multinomial Logistic Regression Model**

| **Variable** | **Coding** |
| --- | --- |
| Sex | 1 = Male; 2 = Female |
| Age group (years) | 1 = 18–34; 2 = 35–49; 3 = 50–65 |
| Educational background | 1 = Primary school or below; 2 = Junior secondary school; 3 = Senior secondary school or vocational school; 4 = College diploma; 5 = Bachelor's degree or above |
| Monthly personal income (yuan) | 1 = <2,000; 2 = 2,000–5,000; 3 = >5,000 |
| Number of comorbidities | 1 = ≤1; 2 = 2; 3 = ≥3 |
| Dialysis duration (months) | 1 = ≤36; 2 = 37–71; 3 = ≥72 |

**Note:** Variables were entered into the multinomial logistic regression model using the numeric codes shown. Sex was coded as a binary variable; age group, educational background, monthly personal income, number of comorbidities, and dialysis duration were entered as ordinal variables.

**Supplementary Table S5. Comparison of Dietary Adherence Behaviour and Attitude Scores Across Nutrition Literacy Profiles Among Patients Receiving PD**

| **Outcome** | **Low profile** **(n = 143)** | **Moderate profile** **(n = 92)** | **High profile** **(n = 72)** | **F** | **FDR-adjusted P** | **η² (95% CI)** | **Bonferroni-adjusted pairwise *P*** |
| --- | --- | --- | --- | --- | --- | --- | --- |
| Dietary adherence behaviour score | 74.97 ± 17.87 | 84.01 ± 16.91 | 86.64 ± 16.00 | 13.943 | <0.001 | 0.084 (0.032–0.146) | L–M <0.001; L–H <0.001; M–H 0.993 |
| Dietary adherence attitude score | 74.55 ± 13.21 | 81.53 ± 13.19 | 88.99 ± 10.93 | 31.847 | <0.001 | 0.173 (0.101–0.247) | L–M <0.001; L–H <0.001; M–H <0.001 |

**Note:** Data are presented as mean ± standard deviation. Overall differences were assessed using one-way analysis of variance. False-discovery-rate-adjusted P values were calculated across the two dietary adherence outcomes using the Benjamini–Hochberg procedure. Pairwise P values were adjusted using the Bonferroni method. Effect sizes are presented as η² with 95% confidence intervals. L, low nutrition literacy profile; M, moderate nutrition literacy profile; H, high nutrition literacy profile; CI, confidence interval; FDR, false discovery rate.
